# Airway Epithelial-Derived Immune Mediators in COVID-19

**DOI:** 10.3390/v15081655

**Published:** 2023-07-29

**Authors:** Tony J. F. Guo, Gurpreet K. Singhera, Janice M. Leung, Delbert R. Dorscheid

**Affiliations:** 1 Centre for Heart Lung Innovation, Providence Healthcare Research Institute, St. Paul’s Hospital, University of British Columbia, 1081 Burrard St., Vancouver, BC V6Z 1Y6, Canada; 2 Department of Medicine, University of British Columbia, 2775 Laurel St., Vancouver, BC V5Z 1M9, Canada

**Keywords:** airway epithelium, COVID-19, cytokines, inflammation

## Abstract

The airway epithelium, which lines the conducting airways, is central to the defense of the lungs against inhaled particulate matter and pathogens such as SARS-CoV-2, the virus that causes COVID-19. Recognition of pathogens results in the activation of an innate and intermediate immune response which involves the release of cytokines and chemokines by the airway epithelium. This response can inhibit further viral invasion and influence adaptive immunity. However, severe COVID-19 is characterized by a hyper-inflammatory response which can give rise to clinical presentations including lung injury and lead to acute respiratory distress syndrome, viral pneumonia, coagulopathy, and multi-system organ failure. In response to SARS-CoV-2 infection, the airway epithelium can mount a maladaptive immune response which can delay viral clearance, perpetuate excessive inflammation, and contribute to the pathogenesis of severe COVID-19. In this article, we will review the barrier and immune functions of the airway epithelium, how SARS-CoV-2 can interact with the epithelium, and epithelial-derived cytokines and chemokines and their roles in COVID-19 and as biomarkers. Finally, we will discuss these immune mediators and their potential as therapeutic targets in COVID-19.

## 1. Introduction

The outcomes and consequences of the coronavirus disease 2019 (COVID-19) pandemic highlight the need to study the molecular mechanisms underlying its pathogenesis in order to develop effective means of diagnosis, treatment, and prevention. COVID-19 has diverse manifestations and varies in severity among infected individuals. Though the majority of infected individuals presents with mild disease characterized by a self-limiting cough, rhinorrhea, malaise, and a sore throat, a portion of patients experience severe respiratory complications, such as dyspnea and hypoxia, requiring hospitalization [1,2]. Some patients can develop COVID-19-associated lung injuries, and these can progress into acute respiratory distress syndrome (ARDS), which is characterized by severe hypoxia and requires oxygen therapy and mechanical ventilation [3]. Diffuse alveolar damage and pulmonary fibrosis can also be long-term outcomes in affected lungs [4]. Progression into severe disease is associated with a dysregulated inflammatory response which can contribute to respiratory and multiorgan failure and result in high morbidity and mortality in hospitalized COVID-19 patients [5,6]. In this context, investigating the airway epithelium, the mucosal and immune barrier lining of the airways, is particularly important. 

The airway epithelium plays a critical role in neutralizing inhaled irritants and pathogens by maintaining a physical barrier against the environment. The airway epithelium is a primary target of SARS-CoV-2 due to the surface expression of viral entry factors [7]. However, the airway epithelium also functions as an immune organ which mediates host immune responses. The epithelial immune response may contribute to severe COVID-19 by initiating inappropriate inflammation with systemic consequences. Thus, characterizing epithelial responses to SARS-CoV-2 is important in understanding viral pathogenesis. In this article, we will review the structure and function of the airway epithelium, consider epithelial-derived cytokines and chemokines and their roles in COVID-19 and as biomarkers, and finally discuss the potential of these immune mediators as therapeutic targets in COVID-19. 

## 2. Main

### 2.1. Airway Epithelial Structure and Function

The respiratory tract can generally be divided into three sections: the upper respiratory tract encompassing the nasopharynx and larynx, the lower respiratory tract comprising the conducting airways, and the respiratory zone comprising the respiratory bronchioles and alveoli [8]. For the purpose of this review, the airway epithelium refers to the lining of the trachea, bronchi, and conducting bronchioles. The airway epithelium is the interface between the environment and the lungs and is the first to encounter inhaled substances. Hence, it helps to prevent the infiltration of environmental insults such as pathogens, particulate matter, and noxious chemicals into the lungs while preventing the leakage of interstitial fluid into the airway lumen [9,10]. The airway epithelium is composed of three major cell types (goblet, ciliated, and basal cells), though recent investigations have revealed that the airway epithelial cellular population is diverse in terms of subtype and differentiation state [11]. Among the rare cell populations in the airway epithelium are tuft and neuroendocrine cells, and these contribute to the stimulation of certain reflexes, such as coughing, and to the augmentation of inflammatory responses, respectively [12,13]. Goblet and ciliated cells contribute to the mucociliary clearance of the airways, whereas basal cells are multipotent stem cells that are important in the reconstitution of the airway epithelium following injury [14]. Histologically, the airway epithelium is pseudostratified, meaning that although the nuclei are located at differing heights, giving the illusion of stratification, all columnar epithelial cells contact the basement membrane [15]. Further down the respiratory tree, the composition of the airway epithelium varies with the airway diameter [16]. Within the small airways, defined as having an internal diameter of ≤2 mm, the epithelium becomes thinner and more columnar, and its airway surface liquid layer becomes thinner [17,18,19]. Within the respiratory bronchioles, the epithelium becomes more cuboidal [19,20]. Though similar cell types are found here, there is a greater representation of secretory club cells, which are multi-functional cells that contribute to detoxification and innate immune responses and function as progenitor cells [21,22]. Accompanying this is a significant reduction in the number of goblet cells as these small-diameter airways are susceptible to luminal blockage [16]. Mucus hypersecretion leading to airway obstruction is observed in some chronic respiratory diseases [23].

**Figure 1:**
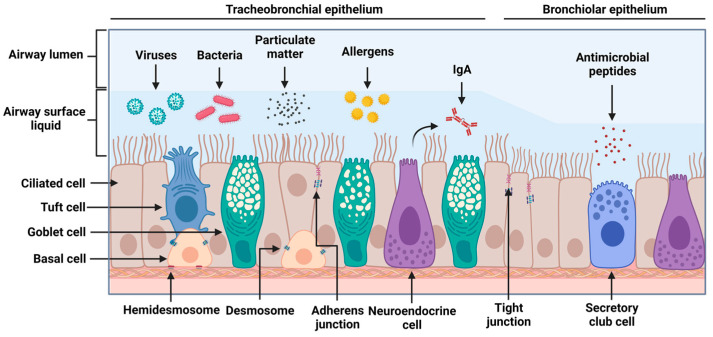
Structure of the airway epithelium. Ciliated, goblet, and basal cells comprise the majority of the cells of the airway epithelium. Among the rare cellular populations are tuft and neuroendocrine cells. The airway surface liquid consists of the mucus and periciliary layers, which are maintained by submucosal glands and goblet cells. The mucus layer traps inhaled particles, such as viruses, bacteria, particulate matter, and antigens, whereas the periciliary layer, which has a lower viscosity, allows ciliary beating. Within the airway surface liquid are antimicrobial factors secreted by the airway epithelium. These include dimeric IgA, which, when bound to its carrier, the polymeric Ig receptor, can undergo transcytosis across the airway epithelium, after which it is released into the apical domain. The tight and adherens junctions that regulate paracellular permeability are present in the apicolateral border of the airway epithelial cells. Desmosomes located near the basolateral surfaces of epithelial cells contribute to cell–cell adhesion and mechanical support. The basal cell connections to the basement membrane are made via hemidesmosomes. These structures contribute to the physical barrier function of the airway epithelium which prevents harmful substances from infiltrating into the submucosa and beyond. As it transitions into the conducting bronchioles, the epithelium becomes more columnar and shorter in height, and its airway surface liquid layer becomes thinner. Furthermore, there is a decrease in goblet cells and a greater proportion of secretory club cells. Figure created in BioRender.com.

The airway epithelium fulfils its function as a physical and immune barrier through numerous means. Junctional complexes, consisting of tight junctions and adherens junctions, are expressed on the apicolateral surfaces of epithelial cells [24]. Tight junctions help to regulate paracellular solute movement, whereas adherens junctions, which are localized below tight junctions, contribute to cell–cell adhesion. Beyond preventing the infiltration of inhaled pathogens through the epithelium, these junctional complexes also contribute to the cell signaling that regulates cell proliferation, differentiation, and the definition of cellular polarity [21,25]. The permeability of this barrier can be increased to allow the infiltration of immune cells into the airway lumen. This is facilitated through two-way communication between the airway epithelium and nearby dendritic cells [26]. Together, they continually sample the airway lumen for potentially noxious substances and determine whether to mount an immune response [27]. The airway epithelium also produces and maintains an airway surface liquid lining that entraps inhaled particles. This lining is composed of a mucus gel layer, which contains mucins and surfactants produced and secreted by goblets cells, and a periciliary layer, which contributes to cilia lubrication. The airway surface liquid lining works in tandem with the cilia of ciliated cells, which beat rhythmically and in a coordinated fashion to move the mucus toward the pharynx so that it can be cleared [28,29]. Lastly, the airway epithelium can constitutively produce antimicrobial peptides, including lysozyme, which degrades the peptidoglycan layer of gram-positive bacteria, and lactoferrin, which sequesters iron to inhibit bacterial growth and binds to viruses itself [30]. Other antimicrobial peptides include cationic molecules such as B-defensins, LL37, and CCL20, which are induced by various pathogens, possess broad-spectrum antimicrobial activity, and contribute to the signaling and recruitment of immune cells [31,32,33]. Plasma cells in the sub-epithelial space can secrete dimeric IgA. The polymeric immunoglobulin receptor (pIgR) binds to dimeric IgA in the basolateral domain of the epithelium to facilitate transcytosis to the apical compartment. The airway epithelium facilitates this process by upregulating the expression of pIgR in response to bacterial ligands [34]. IgA secretion limits microbial infiltration by crosslinking antigens to neutralize and trap them in the mucus layer [35]. Figure 1 depicts the structure and composition of the airway epithelium and its protective functions.

### 2.2. Epithelial Machinery and Secreted Factors in Viral Infections

**Figure 2:**
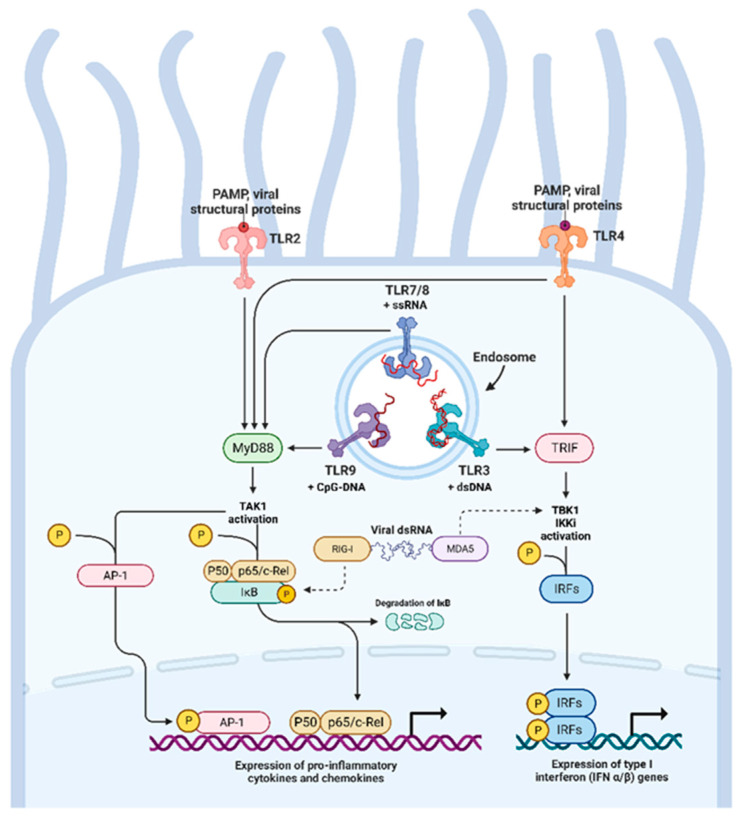
TLR and RLR signaling pathways present in airway epithelial cells in response to viral infections. Viral structural proteins can be detected by TLR2 and TLR4 expressed on the cell surface. Within the endosomes are nucleic acid sensors such as TLR3, 7, 8, and 9. TLR signaling and PAMP recognition can be mediated through MyD88, resulting in AP-1 and NF-κB activation and their translocation into the nucleus to induce the expression of pro-inflammatory mediators. The activation of TLRs can also lead to signaling through TRIF, resulting in the activation of IRFs, which are important in the induction of type I interferon responses. Cytosolic nucleic acid can also be sensed through RLRs such as RIG-I and MDA5, which signal through converging pathways with TLR. Figure adapted from “TLR signaling pathway” and created in BioRender.com.

The airway epithelium encounters inhaled pathogens such as viruses. This tissue possesses innate immune recognition functions which work against these pathogens and allow the induction of an adaptive immune response for effective viral clearance. On the surfaces of airway epithelial cells there are pattern recognition receptors (PRRs) that bind to pathogen-associated molecular patterns (PAMPs). These include Toll-like receptors (TLRs), which can be localized to the plasma membrane and recognize specific moieties such as bacterial lipopolysaccharides and flagellin. Others reside within the lysosomes and endosomes and can detect viral nucleic acids [36]. TLRs include TLR3, which recognizes dsRNA, a viral replicative intermediate, TLR7 and TLR8, which recognize GU and AU-rich motifs within viral ssRNA sequences, and TLR9, which recognizes unmethylated cytosine–guanine dinucleotide motifs [37]. TLR2 and TLR4, which are localized on the plasma membrane, can also recognize viral structural proteins, including the F-fusion protein of the respiratory syncytial virus (RSV), a core protein of hepatitis virus C, and the spike glycoprotein of SARS-CoV-2 [38,39,40]. There is an overlap of downstream signaling pathways across the different TLR families. The recognition of viral PAMPs can lead to the recruitment of the adaptor protein MyD88 and the subsequent activation of the IKK complex–NF–κB pathway and the MAPK pathway, which contribute to the expression of pro-inflammatory factors [41]. TLR activation can also signal through the TRIF-dependent pathway and lead to the activation of interferon regulatory factors (IRFs) such as IRF3 and IRF7. Subsequent phosphorylation of their C-terminal domains by IKK-related kinase complexes containing TBK1 and IKKi leads to the homo- or heterodimerization of the IRFs, their translocation into the nucleus, and their binding to promoters for interferon (IFN) production [42]. Interferons can act in a paracrine and autocrine fashion to upregulate interferon-stimulated genes with broad antiviral functions by interfering with viral mRNA and protein synthesis and strengthening epithelial barrier integrity [43]. MyD88- and TRIF-dependent TLR signaling can activate NF-κB. IkB kinase complexes can also liberate NF-κB through IκBα polyubiquitylation and proteasomal degradation, leading to NF-κB nuclear translocation and the transcription of pro-inflammatory cytokines. Retinoic acid-inducible gene I-like receptors (RLRs), such as RIG-I and melanoma differentiation-associated protein 5 (MDA5), are expressed in airway epithelial cells. These are cytoplasmic RNA helicases that undergo RNA binding-dependent conformational changes, and which, with adaptor proteins, activate IKK, TBK1, and NF-κB [44]. The induction of type I and III interferons and interferon-stimulated genes is a central feature of type 1 epithelial immunity, and it is important in the response to intracellular pathogens such as viruses. Other responses include type 2, which is involved in parasite and fungal infections, and type 3, which is involved in controlling extracellular bacteria and fungi [45]. Figure 2 illustrates the TLRs and other PRRs that exist within airway epithelial cells in response to viral infections. 

During stress, the airway epithelium releases alarmin cytokines which can promote adaptive immune responses and tissue repair. These include thymic stromal lymphopoietin (TSLP), IL-25, and IL-33, which promote type 2-polarized immune responses. The release of these alarmins activates resident dendritic cells and innate lymphoid 2 cells (ILC2s). Activated dendritic cells then migrate to the lymph nodes to induce the activation and differentiation of effector CD4^+^ Th2 cells. This ultimately produces cytokines associated with type 2 immunity, such as IL-4, IL-5, IL-9, and IL-13 [46,47]. These cytokines promote IgE class-switching in B-cells and eosinophil activation and recruitment, and they induce goblet cell hyperplasia, mucus hypersecretion, and airway reactivity [48]. Type 2 cytokines are also implicated in allergic diseases such as chronic rhinosinusitis, asthma, and atopy [49].

TSLP binds to a heterodimer receptor complex of the IL-7 receptor alpha chain and the TSLP receptor, expressed in innate and adaptive immune cells. The highest expression of the TSLP binding receptors is found in dendritic cells [47]. TSLP regulates airway inflammation by activating dendritic cells to express the OX40 ligand (OX40L), which differentiates naïve CD4^+^ T cells into Th2 polarized T cells, producing IL-4, IL-5, and IL-13. Th2 T cells also activate naïve CD8^+^ T cells, which subsequently produce IL-5 and IL-13. TSLP promotes eosinophil survival through greater adhesion to fibronectin, and it also encourages the release of IL-6, CXCL1, CXCL8, and CCL2, which promote the migration of neutrophils and non-hematopoietic cells [47]. 

IL-33 is expressed at basal levels in the airway epithelium, but its expression and secretion can be upregulated during viral infections [50]. IL-33 can also be released passively in the extracellular space through necrotic cell death [51]. This alarmin can bind to its receptor suppression of tumorigenicity 2 expressed on immune cells involved in innate and adaptive immunity to stimulate the production of IL-4, IL-5, and IL-13 [50]. IL-33 promotes the degranulation of eosinophils and histamine release in mast cells and upregulates the production of cytokines and chemokines such as IL-6, TNF, IL-1β, and CCL17 in dendritic cells. Like TSLP, IL-33 can increase the expression of co-stimulatory molecules, OX40L, and MHC class II molecules in dendritic cells, all of which promote T cell proliferation and differentiation [52]. IL-33 can also act on ILC2 in the blood and in mucosal tissues to promote the expansion and production of IL-5 and IL-13 [53]. 

IL-25 is expressed by airway endothelial and epithelial cells, mast cells, Th2 cells, basophils, activated eosinophils. This cytokine binds to a heterodimer of IL-17RA and IL-17RB found on mast cells and eosinophils [54]. The binding of the cytokine to the receptor results in a signaling cascade through TRAF4 and TRAF6, and in subsequent gene expression changes through NF-κB. The release of IL-25 can subsequently trigger the overproduction of IL-4, IL-5, IL-9, IL-13, and CCL11 from differentiated Th2 cells and epithelial cells [55]. It can also induce the release of cytokines and chemokines such as IL-6, IL-8, MCP-1, and MIP-1α in eosinophils, and these perpetuate neutrophilic inflammation and accelerate monocyte recruitment within the airways [56]. IL-25, like other epithelial alarmins, plays an important role in airway remodeling, a phenomenon characterized by the thickening of the airway wall, goblet cell hyperplasia, and increased angiogenesis [57]. IL-25 can act directly on fibroblasts to induce collagen secretion and recruit endothelial progenitor cells to contribute to angiogenesis [58].

Other pro-inflammatory cytokines and chemokines can be released in response to PRR activation in the airway epithelium. These include IL-6, IL-8, CCL20, granulocyte-macrophage colony-stimulating factor (GM-CSF), IL-1α, IL-1β, and TNF-α, which are released in response to RSV and influenza virus infections [59,60]. These can increase cellular recruitment into the infected airways, promoting injury to the infected and uninfected lung tissue. In the context of RSV infections, autocrine signaling through the airway epithelial release of TNF-α, IL-1α, and IL-1β can upregulate the release of IL-6, Il-8, and CCL5, which facilitate the recruitment of immune cells, including neutrophils and monocytes, which then exert their antiviral effects [61]. Neutrophils play an important role during viral infections [62]. They induce the release of reactive oxygen species and the subsequent release of neutrophil elastase, which degrades antigens, causing tissue damage and extruding genomic material to form extracellular traps to confine viruses [63,64]. Airway macrophages are responsible for the phagocytosis of pathogens and for antigen presentation, which can mediate the recruitment of other immune cells. These can include monocyte-derived macrophage cells that, after recruitment, can translocate into the airway luminal niche by migrating in a transepithelial manner [65]. During viral infections, these macrophages also produce and respond to cytokines that contribute to the priming of the macrophage for antiviral activity and tissue repair [65,66].

### 2.3. COVID-19, SARS-CoV-2, and the Airway Epithelium

Severe acute respiratory syndrome coronavirus 2, or SARS-CoV-2, is responsible for COVID-19. It is a positive sense single-stranded RNA virus closely related to other human coronaviruses, such as HCoV-229E and HCoV-OC43, which cause seasonal and usually mild upper respiratory tract infections [67]. The large 30 kilobase pair genome of SARS-CoV-2 encodes for 31 proteins. These include structural proteins, non-structural proteins that are involved in viral replication and transcription, and accessory proteins that are involved in evading innate immune responses. The structural proteins include the components for the spike, envelope, membrane, and nucleocapsid. Among the accessory proteins are ORF3a, ORF3b, ORF6, ORF7a, ORF7b, ORF8, ORF9b, and ORF10 [68].

SARS-CoV-2 enters susceptible host cells by binding its spike glycoprotein, located on the surface of the viral membrane, to angiotensin-converting enzyme 2 (ACE2) [69]. The proteolytic cleavage of the viral spike protein is required to facilitate the membrane fusion, which is mediated by TMPRSS2 on the cell surface or by cathepsin B and L in the endosomes if the virus has been endocytosed. The expression of ACE2, TMPRSS2, and other host viral entry factors defines the viral tropism of SARS-CoV-2 [70].

ACE2 and TMPRSS2 are expressed within the bronchial epithelium, primarily in ciliated cells, and are absent in goblet cells at both the mRNA and protein levels [71]. The susceptibility of the airway epithelium to SARS-CoV-2 infection has been demonstrated by the detection of SARS-CoV-2 in bronchoalveolar lavage and tracheal aspirates [72,73]. Furthermore, the in situ expression of SARS-CoV-2 has been observed in airway and lung biopsies from autopsies of individuals with COVID-19, primarily localized to ciliated cells and alveolar pneumocytes [74]. ACE2 is also expressed in the epithelium of the small and large airways and trachea, though its expression is significantly lower in the small airway epithelium than in the trachea or large airways [7]. Active SARS-CoV-2 infection has also been demonstrated in in vitro models of the airway epithelium. Submerged monolayer cultures of airway epithelial cells lines, such as BEAS-2B and Calu-3, have been used to characterize interferon responses and viral entry inhibition [75,76]. However, differentiated three-dimensional air–liquid interface (ALI) cultures of primary airway epithelial cells are more relevant models. These cultures can more closely mimic the structure and function of the in vivo epithelium and capture the biological variation in the population. This is especially important as the expression of ACE2, susceptibility to COVID-19, and clinical outcomes are different in certain demographic groups and in different disease processes [77,78]. ALI cultures are readily infected with SARS-CoV-2, and the release of cytokines and chemokines is induced [79,80,81,82]. Do et al. (2022) observed SARS-CoV-2 replication in ALI cultures of bronchial and small airway epithelial origins, though a greater proportion of the bronchial cultures were infected when exposed to SARS-CoV-2 isolates compared with the small airway epithelial cultures. Furthermore, infected bronchial and small airway epithelial cultures produced CXCL10, a chemokine, and Il-6, though no statistical comparisons have been made between the two types of cultures [83]. Comparable gene expressions of SARS-CoV-2 entry factors, including ACE2, TMPRSS2, and CTSL (a lysosomal protease), have been observed in submerged and differentiated cultures of bronchial and small airway epithelial cells [84]. SARS-CoV-2 infection induces apoptosis and syncytia formation, and it also inhibits cilia function in primary airway epithelial ALI cultures [85]. These results demonstrate that the airway epithelium expresses critical proteins that are necessary for viral entry and that can facilitate active infection with SARS-CoV-2.

### 2.4. Epithelial-Derived Immune Mediators in SARS-CoV-2 Infections and Their Roles as Biomarkers

For most patients, the airway epithelial innate immune response is sufficient to clear the virus, and this is followed by a subsequent recession of the immune response. As airway epithelial cells are one of the first cell types to sense respiratory pathogens, they must communicate and coordinate with nearby cells, including immune and endothelial cells, to control viral propagation and subsequently facilitate the resolution of the inflammatory response. This bilateral intercellular communication in response to a stimulus, which is termed “cross-talk”, can occur through physical cell–cell contact or the secretion of immune mediators [8,86]. In severe COVID-19, lung injury may result from a hyperactivated immune system and inappropriate interactions between the immune system and the airway epithelium. Chua et al. (2022) performed single-cell RNA sequencing on bronchial samples from patients with moderate or critical disease to characterize mucosal immune responses [87]. In critical COVID-19 patients, they observed increased expressions of chemokines and cytokines as well as epithelial–immune cell interactions that are consistent with a higher activation of non-resident and monocyte-derived macrophages and cytotoxic T cells. They also observed that ACE2 expression increased in a subset of epithelial cells following SARS-CoV-2 infection, and this correlated with the number of ligand–receptor interactions involving cytotoxic T lymphocytes, likely due to *IFNG* signaling between epithelial and immune cells [87]. The immune-mediated upregulation of the SARS-CoV-2 viral receptor may exacerbate the airway epithelial inflammatory response by perpetuating viral entry and infection. Furthermore, Thorne et al. (2021) explored the link between epithelial infection and macrophage inflammation by treating macrophages with conditioned media of SARS-CoV-2-infected Calu-3 airway epithelial cells. They observed that macrophage inflammation is induced by the recognition of SARS-CoV-2 by RIG-I/MDA5 systems in epithelial cells, and that this effect is exacerbated if there is pre-existing immune activation of the macrophages via bacterial lipopolysaccharide stimulation. By blocking RNA sensing in the infected airway epithelium, the stimulatory activity of the conditioned media and the macrophage activation is reduced by inhibiting epithelial inflammatory gene expression [88]. The crosstalk between the airway epithelium and the immune system is critical in propagating epithelial damage or a pro-inflammatory response in COVID-19.

Beyond interactions in the epithelial–immune axis, the infected epithelium can also damage the endothelium. Deinhardt-Emmer et al. (2021) used a human chip model containing epithelial and endothelial cells to characterize epithelial and endothelial barrier function following SARS-CoV-2 infection. It was observed that the endothelial cells were not successfully infected despite being cultured near a SARS-CoV-2-infected epithelium. However, the endothelium was damaged, and its barrier function decreased as the infection time increased, likely because of the release of cytokines from the damaged epithelium [89]. Reportedly, SARS-CoV-2 can also indirectly induce glycocalyx damage and an endothelial immune response [33,90]. A reduction in endothelial barrier integrity would increase vascular permeability and thereby contribute to COVID-19 viremia and the systemic dissemination of the virus. Treatment of endothelial cells with the viral spike glycoprotein can directly activate endothelial cells and cause them to degrade junctional proteins and secrete inflammatory molecules [91,92]. As endothelial cells express minimal levels of ACE2 [93], this mechanism is likely a result of PAMP recognition by PRRs. Cytokines released following SARS-CoV-2 infection of the airway epithelium and present within the extracellular milieu can also induce endothelial dysfunction and additional cytokine release from the endothelium in an indirect paracrine manner [94]. These observations highlight the importance of the airway epithelium as a central mediator for the direct and indirect mechanisms of SARS-CoV-2-induced endothelial dysfunction. Following productive replication in the airway epithelium, viruses, PAMPs, and cytokines are shed and secreted. Endothelial cells, which are generally resistant to SARS-CoV-2 infection, respond to PAMPs and cytokines which disrupt their function and amplify inflammatory factor and mediator release. Therefore, inappropriate or exaggerated interactions between the airway epithelium and nearby endothelial cells and the immune system can contribute to the hyperinflammatory cascade and the vascular pathologies seen in severe COVID-19. 

During the acute response to viral infections, IFNs secreted by the airway epithelium play a central role in coordinating an antiviral immune response. SARS-CoV-2 infection and replication are recognized by TLR3 and TLR7 [95], which bind viral RNA and are localized within the endosome, and by RIG-I and MDA5, which are localized within the cytosol [88]. Signal transduction through cytosolic viral RNA sensors occurs via the mitochondrial antiviral-signaling protein (MAVS). However, the SARS-CoV-2 spike protein can bind to membrane-expressed TLR2 to induce an inflammatory response in airway epithelial cells [96]. Following the recognition of PAMPs, transcription factors translocate to initiate the cellular antiviral response. However, with SARS-CoV-2 infection, type I and III IFN responses are dampened compared with the responses to other viruses, which is suggestive of immune invasion. A study of 54 patients hospitalized with COVID-19 and respiratory failure, the patients showed significantly lower blood IFN-α and IFN-β levels at recruitment. However, in survivors, and with improvements in disease severity, this was associated with an increase in blood IFN-α [97]. Furthermore, plasma levels of IFN-α were significantly lower in critical COVID-19 patients than in patients with mild or moderate COVID-19 [98].

SARS-CoV-2 accessory proteins can also contribute to immune invasion. ORF9b can inhibit the interaction between RIG-I and MAVS, and the ORF7a protein destabilizes TBK1, reducing IRF3 phosphorylation and IFN expression [99]. ORF6 can also interact with Nup68, a nucleoporin complex protein, disrupting nuclear transport and STAT1 and STAT2 translocation and thereby inhibiting interferon-stimulated genes [100]. Other mechanisms by which SARS-CoV-2 viral proteins antagonize IFN responses are reviewed by Lee et al. (2022) [101]. The correlation of type I IFN with disease status is poor. A systematic review of 15 studies investigating the association of plasma protein levels of type I IFN with the severity of COVID-19 found no significant difference when comparing mild and severe patients [102].

The airway epithelium also produces a myriad of pro-inflammatory mediators which have been observed to be upregulated with COVID-19. In the same study by Chua et al. (2020), the authors observed a higher expression of chemokine genes involved in neutrophil, T cell, and mast cell recruitment in goblet cells, and of chemokines involved in monocyte and neutrophil recruitment in ciliated cells. Recruited macrophages then express pro-inflammatory mediators, contributing to further monocyte recruitment [87]. Transcriptome profiling of in vitro models of the airway epithelium revealed increased expressions of IL-1β, TNF-α, CCL2, CCL20, CCL8, CXCL2, CXCL8, and CXCL16 (all of which are upregulated in severe COVID-19), resulting in lung injury and multiorgan failure [82,89,103,104]. IL-6 has been identified as an important biomarker for SARS-CoV-2 infection and COVID-19 progression. Barnett et al. (2023) studied the dynamic of IL-6 secretion in COVID-19 using SARS-CoV-2-infected airway epithelial cells co-cultured with peripheral blood mononuclear cells. They observed that the epithelium released damage-associated molecular patterns (DAMPs) that stimulate leukocytes to produce IL-1β. The paracrine function of IL-1β involves stimulating both leukocytes and epithelial cells to produce IL-6, with the latter cell type secreting higher amounts, thus creating a feedback circuit. This process requires inflammasome activation within leukocytes, which only occurs following the release of DAMPs from the injured epithelium [105]. These results highlight the importance of the airway epithelium in kickstarting the initial inflammasome activation through viral infection, and in kickstarting DAMP release and cytokine release through epithelial–immune crosstalk. In a parallel pathway, SARS-CoV-2 infection can also amplify IL-6 secretion by the airway epithelium. Upon binding to ACE2, the virus is endocytosed, which downregulates the surface expression of ACE2. A reduction in ACE2 increases the level of serum angiotensin II (AngII) (the substrate for ACE2) and the level of signaling through its cognate receptor, AT1R. ACE2 is also downregulated in models of acute lung injury and ARDS, which suggests that a dysfunctional renin–angiotensin system plays a role in the pathogenesis of the disease [90]. AngII–AT1R axis signaling induces NF-κB and ADAM17 expression, and the latter cleaves IL-6Ra into its soluble form, which can activate the JAK/STAT3 pathway in epithelial cells [106]. This induces the activation of a positive feedback loop called the IL-6 amplifier (IL-6 Amp), whereby the activation and nuclear translocation of STAT3 upregulate the release of pro-inflammatory cytokines including IL-6 [107]. The airway epithelium, therefore, can initiate IL-6 production following SARS-CoV-2 infection through immune crosstalk, but it can also sustain its release through the AngII/AT1R pathway, which may contribute to excessive IL-6 release. IL-6 is a biomarker for fatal pneumonia and COVID-19 severity [108,109], and this has been reviewed by Liu et al. (2020) and Sebbar and Choukri (2023) [108,110].

IL-33 has been investigated as a marker in the pathogenesis and prediction of severe COVID-19. Liang et al. (2021) infected two epithelial cell lines, FaDu and LS513, with SARS-CoV-2. They observed significantly higher IL33 expression at 72 h post-infection, demonstrating that infection induces the expression of IL-33 in the airway epithelium [111]. RNA sequencing of bronchoalveolar lavage fluid from COVID-19 patients also revealed significantly increased IL33 expression [112]. Gaurav et al. (2021) characterized the expression of IL-33 in post-mortem lung sections of patients with COVID-19. They observed that five of the eight lungs had IL-33 expression, but at low levels due to release and depletion. The authors suggest that the lung compartment is an important source of IL-33 [113]. Within the lungs, the airway epithelium is a primary reservoir for IL-33. Byers et al. (2013) demonstrated that IL-33 expression was localized in airway serous cells and alveolar type 2 cells in mice. In humans, IL-33 expression is localized in the nuclei of airway basal progenitor cells, and this increases in chronic respiratory diseases [114]. During SARS-CoV-2 infection, IL-33 is released by the airway epithelium, and it may leak into systemic circulation and be detected. Investigations have revealed that plasma IL-33 is elevated in COVID-19 patients and that it is predictive of disease severity. Markovic et al. (2021) analyzed the serum of 220 COVID-19 patients who had been stratified into 2 groups based on severity. They observed that IL-33 was significantly elevated in patients with severe disease and that it correlated with clinical features and radiographic findings. Similarly, Burke et al. (2020) performed multiplex cytokine assays to measure serum cytokine levels in hospitalized COVID-19 patients. They observed that in patients ≤70 years old, serum IL-33 and TNF-α were predictive of poor outcomes [115]. IL-33 may contribute to the pathogenesis of severe COVID-19 through dampening antiviral responses and through contributing to lung fibrosis, neutrophil migration, thromboses, and mast cell and eosinophil activation, all of which has been reviewed by Murdaca et al. (2022) and Zizzo and Cohen (2020) [50,116]. Adding to this complexity is the fact that IL-33 participates in antiviral responses and the resolution of inflammation. IL-33 can contribute to type 1 immunity by expanding CD8^+^ T cells and causing them to produce effector cytokines such as IFN-γ by potentiating IL-12-mediated T cell differentiation [117,118]. Following adequate viral clearance, IL-33 promotes inflammation resolution by enhancing the differentiation of regulatory T cells. These cells participate by suppressing γδ T cells, which are activated in response to tissue infection, and promoting tissue repair following injury [119,120]. In severe COVID-19, the overproduction of IL-33 may accelerate the polarization and activation of ILC2. This in turn can prolong the survival of γδ T cells and suppress NK cell innate immunity [111,116,121]. Because it is a cellular alarmin indicating damage and infection, extensive damage of the airway epithelium may contribute to the excessive release of IL-33 and progression toward severe disease.

TSLP can be released by airway epithelial cells in response to the presence of viral markers. Kato et al. (2007) stimulated normal human bronchial epithelial (NHBE) cells with dsRNA, IL-4, and IL-13. They observed that signaling through IRF-3 using TLR3, the agonist of dsRNA, induced TSLP production, which implies that respiratory viral infection induces TSLP production [122]. Gerla et al. (2023) cultured NHBE cells and patient bronchoscopy-derived primary human bronchial epithelial cells and infected them with SARS-CoV-2. They observed that intracellular and secreted TSLP expression significantly increased with SARS-CoV-2 infection. Increased TSLP expression also correlated with increased viral titers. To validate their findings, the authors collected plasma samples from COVID-19 patients, but they observed no significant difference between the plasma cytokine levels of TSLP in the control patients and those in the hospitalized patients. However, higher plasma TSLP levels at the time of hospitalization were associated with an increased hospitalization period [123]. In contrast, it has been observed that TSLP can promote airway epithelial homeostasis. TSLP treatment increases airway epithelial cell proliferation and wound closure following injury [124]. Blocking TSLP in mice with bleomycin-induced ARDS increases inflammation and morbidity. Furthermore, the treatment of bleomycin-treated NHBE cells with TSLP promoted survival by upregulating Bcl-xL and reducing caspase 1 and 3 activity [125]. Considered together, these findings suggest that the deleterious effects of epithelial-secreted TSLP in COVID-19 may occur downstream of the epithelium.

IL-25 has been implicated in viral-induced inflammation. Petersen et al. (2014) infected mice deficient in IL-17RB (the receptor for IL-25) with RSV and observed a reduction in pulmonary cytokine transcripts, mucus hypersecretion, and improved viral clearance [126]. Beyond inhibiting lung pathology associated with IL-25, blocking IL-25 is also helpful in restoring antiviral immunity. Williams et al. (2022) infected primary air–liquid interface cultures with both rhinovirus and hCoV229E coronavirus strains. They observed that treatment with an IL-25-blocking monoclonal antibody significantly increased IFN-y secretion with coronavirus infection, and they also observed a trend of upregulation in IFN I/III secretion, though this was insignificant. In the context of rhinovirus infection, suppressing IL-25 promoted IFN I/III- and interferon-stimulated gene expression, improved antiviral innate immunity, and reduced viral load [127]. Ullah et al. (2022) analyzed the expression of IL25 in peripheral blood mononuclear cells from infected COVID-19 patients. They observed that IL25 was significantly elevated in COVID-19 patients, though specific findings related to IL-25 and its contribution to the COVID-19 inflammatory response or to mortality were not discussed [128].

IL-33, TSLP, and IL-25 are released by the epithelium to promote type 2 immunity, which can inhibit type 1 responses that are important in the defense against viral infections. The balance between type 1 and type 2 immune responses has been linked to clinical outcomes in COVID-19 cases. Individuals who recovered from mild COVID-19 had a robust type 1 response associated with effective viral clearance and the resolution of infection [129]. However, type 2 immune responses were more frequent in patients requiring intensive care [130]. Furthermore, in COVID-19 patients, the percentages of Th1 and Th17 cells were significantly reduced, and the percentage of Th2 cells was increased. Senescent Th2 cell percentage was also an independent risk factor for mortality [131]. Immune profiling of patients with moderate COVID-19 revealed a reduction in type 1 and type 3 responses. However, severe COVID-19 patients more frequently had type 2 responses characterized by an increase in type 2 cytokines, immunoglobulin E, and eosinophils [132]. An effective initial type 1 response early in SARS-CoV-2 infection is essential for cell-mediated immunity against the virus. However, a bias toward type 2 inflammation may lead to a delayed antiviral response, and it is associated with increased severity of COVID-19. Table 1 presents the selected immune mediators secreted by the airway epithelium and summarizes their role in COVID-19.

IL-4 and IL-13 are important type 2 inflammation cytokines produced by CD4^+^ T cells, basophils, eosinophils, mast cells, and stimulated ILC2 cells, whose production is induced through IL-33, TSLP, and IL-25 signaling [48,133]. In mice infected with influenza virus, IL-4 treatment inhibits cytotoxic T cell responses and inhibits viral clearance [134]. Accordingly, higher levels of IL-4 in the sera of COVID-19 patients positively correlates with both the time to viral clearance and severe illness [135]. Elevated levels of serum type 2 cytokines, including IL-4 and IL-13, were found to be predictive of life-threatening COVID-19 in hospitalized patients [136]. Furthermore, plasma IL-4 levels were found to be elevated in young, comorbidity-free COVID-19 patients in an intensive care unit [137]. These cytokines are also important in pathological responses in the lung. They are associated with the induction of airway mucus secretion, goblet cell metaplasia, smooth muscle cell proliferation, and airway fibrosis due to fibroblast activation and collagen deposition [138,139]. The tissue expressions of IL-4 and sphingosine-1, a marker for M2 subtype macrophages, were found to be significantly increased in post-mortem lungs from COVID-19 patients compared with those from patients infected with influenza virus and those of control patients [140]. IL-4 and IL-13 can activate M2 macrophages, causing them to induce a repair response characterized by the expansion of resident fibroblasts and the proliferation of airway progenitor cells. This could contribute to the remodeling and fibrosis of the lungs seen with acute respiratory disease syndrome and accompanied by diffuse alveolar damage [141].

**Table 1:** Selected immune mediators secreted by the airway epithelium and summaries of their roles in COVID-19.

Immune Mediators Upregulated or Secreted in the Airway Epithelium in Response to SARS-CoV-2 Infection and COVID-19	Role in COVID-19
Chemokines CXCL1, CXCL3, CXCL6, CXCL17 [87] IL-8 [89,142] CXCL2 [143]	Recruits monocytes, macrophages, T cells, neutrophils [104] Contributes to immune cell-mediated lung tissue damage [144,145]
Pro-inflammatory cytokines IL-1β [89,146] IL-10 [146,147] IL-6 [89,105,142] TNF-α [89,142]	Induction of acute phase protein response by hepatocytes [104] Elevated levels of these cytokines are associated with respiratory distress syndrome and severe disease [148]
Epithelial-derived alarmins IL-33 [111] TSLP [123]	Promotion of type 2 inflammatory response and inhibition of type 1 responses; associated with requirement for intensive care [129,130]

**Figure 3:**
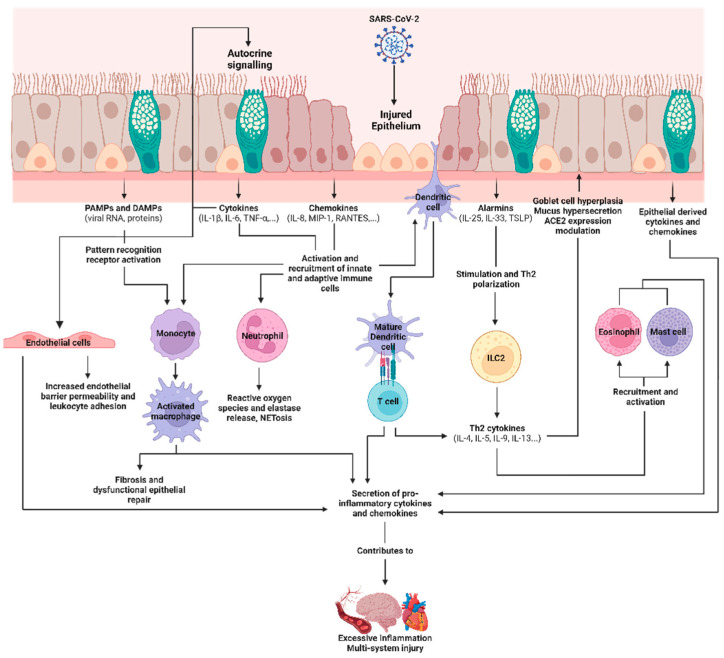
Epithelial immune mediators released during SARS-CoV-2 infection and their roles in the progression of COVID-19. The airway epithelium expresses host viral entry factors such as ACE2, TMPRSS2, and furin, and these facilitate the binding and entry of SARS-CoV-2. Following entry, replication can occur, generating and shedding viral PAMPs, which, with cellular injury, can also cause the release of DAMPs from the epithelium. Immune cells, including monocytes, can sense these factors, resulting in macrophage differentiation. The generation of PAMPs within the airway epithelium is sensed by PRRs which trigger signaling responses to upregulate an antiviral response characterized by the secretion of cytokines, chemokines, and epithelial alarmins. These mediators recruit and activate immune cells, including non-resident monocytes and neutrophils. Resident dendritic cells are stimulated by viral PAMPs and migrate to the lymph nodes to induce the differentiation of T cells, contributing to cytokine release. Epithelial alarmins can contribute to the type 2 polarization of T cells and innate lymphoid cells to produce type 2 inflammation cytokines. These contribute to mucus hypersecretion, goblet cell hyperplasia, and the downregulation of ACE2 expression within the airway epithelium, which modulates susceptibility to SARS-CoV-2. Granulocytes are also recruited to the lungs. PAMPs and cytokines released by the epithelium can also stimulate the nearby endothelium to increase permeability, leukocyte adhesion, and the production of proinflammatory cytokines. The immune feedback between the airway epithelium, immune cells, and endothelium can lead to the over-production of cytokines and chemokines, which contributes to excessive systemic inflammation and multi-system injury in severe COVID-19. Figure created in BioRender.com.

The airway epithelium can secrete IL-13, which is an important mediator involved in repair following injury [115,116]. Plasma IL-13 levels were found to be significantly elevated in COVID-19 patients with severe disease and those requiring mechanical ventilation. Furthermore, in a murine model of SARS-CoV-2 infection, there was significant enrichment in the genes involved in IL-4 and IL-13 signaling and increased hyaluronan deposition in the parenchyma of the lungs. These observations were attenuated with IL-13 neutralization [149]. However, there have been reports that IL-13 is protective against epithelial damage resulting from SARS-CoV-2 infection. Pre-treatment of in vitro airway epithelial cultures with IL-13 has been found to reduce cell shedding and viral titers, likely because of the physical barrier produced by mucus hypersecretion. This effect was reduced by removing the mucus or by using knockout cells of MUC5AC, a major component in mucus [150]. However, CRISPR-Cas9 targeting of SPDEF, which controls MUC5AC and MUC5B expression in goblet cells and is induced by IL-13 signaling, did not change viral titers when compared with a non-targeting control, indicating that the antiviral effects of IL-13 may be independent of SPDEF and MUC5AC induction [151]. Figure 3 illustrates the roles of airway epithelial immune mediators in SARS-CoV-2 infection and the progression of COVID-19, and their involvement with viral entry factors, and in viral replication, immune cell activation, cytokine release, and systemic inflammation.

### 2.5. Current and Future Strategies for COVID-19 Therapy

Therapeutic approaches to managing severe COVID-19 include the inhibition of viral replication, broad immunosuppression, oxygen therapy, and supportive care. Antivirals, such as remdesivir, are recommended for patients hospitalized with COVID-19, with or without minimum oxygen therapy. Managing excessive inflammation and coagulopathies that contribute to hypoxemia takes priority as disease severity progresses, and this necessitates more aggressive oxygen interventions. Thus, glucocorticoids, such as dexamethasone, and prophylactic or therapeutic doses of anticoagulants such as heparin, are administered [1,152]. Dexamethasone was particularly effective in reducing mortality for hospitalized COVID-19 patients requiring mechanical ventilation or oxygen therapy, but it was detrimental in those not receiving respiratory support [153]. The administration of dexamethasone to critical COVID-19 patients was found to significantly reduce the plasma levels of inflammatory mediators including IL-1β, IL-6, IL-8, IL-10, and MIP-1α, and this to counteracted hyper-inflammation [154].

Immunomodulatory therapies that are more targeted than steroids have been investigated for their ability to reduce inflammation by targeting pro-inflammatory cytokines and their signaling pathways. Because an elevation of IL-6 has been identified in severe cases of COVID-19, monoclonal antibodies that block IL-6 receptor signaling, such as tocilizumab, have been tested. Initial observations in patients with severe and critical COVID-19 to whom tocilizumab was administered showed improvements in clinical presentations and laboratory examinations, and all of these patients were discharged within an average of 15 days [155]. Subsequent trials which aimed to replicate the larger randomized clinical trial found little to no benefit in terms clinical endpoints or mortality [156,157,158]. However, these trials differed in terms of their size, study design, and the disease characteristics of the enrolled participants. The RECOVERY randomized open-label controlled trial enrolled 4116 hospitalized COVID-19 patients to assess tocilizumab, and this represents the largest trial of tocilizumab to date. The trial reported that patients allocated tocilizumab had a higher likelihood of being discharged within 28 days as well as a decreased likelihood of requiring invasive mechanical ventilation and death when compared with patients receiving usual care [159].

IL-1 antagonists, such as anakinra, have also been investigated for their ability to treat the hyperinflammatory phase of COVID-19. Anakinra is primarily used to treat rheumatoid arthritis, though its effectiveness in treating pericarditis has been investigated [160,161]. Barkas et al. (2021) performed a meta-analysis of nine studies involving a total of 1119 hospitalized COVID-19 patients recruited in trials intended to evaluate the effect of anakinra treatment on the requirement for invasive mechanical ventilation and on mortality. Pooled together, anakinra reduced both mortality and the need for mechanical ventilation when compared with standard care, and no differences in the risk of adverse effects were observed [162].

Targeting airway inflammation and modulating the release of epithelial-derived cytokines using inhaled corticosteroids (ICSs) can reduce the secretion of epithelial-derived cytokines and chemokines. ICSs remain a mainstay therapeutic in the management of asthma, a disease characterized by chronic airway inflammation, airway hyperresponsiveness, and epithelial alterations such as goblet cell hyperplasia and sub- and intra-epithelial inflammatory cell accumulation [163]. Glucocorticoids exert their anti-inflammatory effect within the airways by suppressing inflammatory leukocyte migration and activation through the induction of the apoptosis of eosinophils and T cells [164]. However, the airway epithelium is also responsive to corticosteroid treatment. Treatment of the airway epithelium with ICSs such as fluticasone and budesonide can reduce the expression of proinflammatory genes, such as cytokines and chemokines [165,166,167]. Budesonide treatment can inhibit the secretion of TSLP and eotaxin-3 (a chemoattractant of eosinophils and basophils) from rhinovirus-infected or polyinosinic:polycytidylic acid-stimulated NHBE cells [168]. In addition, it can also suppress IL-6, IL-8, CCL5, and CXCL10 release from the BEAS-2B airway epithelial cell line in a dose-dependent manner following rhinovirus infection [169]. Budesonide can attenuate the barrier disruption caused by dsRNA treatment in in vivo murine and in vitro airway epithelial models [170]. It therefore may further reduce the infiltration of viral PAMPs and sensitization by sub-epithelial immune cells. In mice, fluticasone treatment significantly downregulated ACE2 protein and mRNA expression at the airway epithelium [171]. Reducing surface ACE2 viral receptors may augment SARS-CoV-2 susceptibility, though SARS-CoV-2 infection assays have not been performed to confirm this. Pre-treatment of human airway epithelial cells with a combination of therapeutics commonly used in managing asthma exacerbations has also been found to reduce viral titers and the production of cytokines following HCoV-229E infection. This combination included a muscarinic antagonist, a β2 agonist, and budesonide [172]. Other ICSs, such as ciclesonide, can also inhibit the replication of SARS-CoV-2 in cultured human airway epithelial cells by targeting the formation of the double-membrane vesicles that are necessary for viral replication [173]. ICSs, especially budesonide, have been studied for their ability to treat patients with mild COVID-19 and those at high risk for severe COVID-19. In the STOIC randomized clinical trial, the inhalation of budesonide led to a relative reduction of 91% in clinical deterioration. It reduced the time to recovery and the likelihood of needing urgent medical care after early COVID-19 [174]. In the PRINCIPLE clinical trial, inhaled budesonide reduced the likelihood of hospital admission or death and reduced the time to recovery in patients with COVID-19 [175]. A combination of oral fluvoxamine, a selective serotonin reuptake inhibitor, and inhaled budesonide also reduced the incidence of severe COVID-19 among high-risk outpatients with early COVID-19 [176]. ICSs may exert their beneficial effects by reducing epithelial inflammation and viral propagation and thereby mitigating excessive downstream recruitment and activation of the immune system. This may explain why prophylactic or adjunctive administration of ICSs can reduce the likelihood of progression toward severe disease in COVID-19 patients.

Epithelial alarmins may be beneficial therapeutic targets that could help inhibit the progression toward severe airway and systemic inflammation in COVID-19 patients. Clinical trials and pre-clinical experimental studies of biologics targeting IL-33, TSLP, IL-25, and cytokines such as IL-4, IL-5, and IL-13 have been undertaken to determine their ability to treat severe asthma [177,178,179,180]. Desvaux et al. (2021) conducted a computational network analysis to identify potential therapeutic targets by profiling SARS-CoV-2-infected epithelial and endothelial cell databases. They identified drugs targeting IL-1β, IL6, IL-6Rα, and TNF-α, as well as corticosteroids, through interactome mapping between COVID-19-related proteins and their known drug targets according to current therapeutic approaches. However, they also identified alarmins, such as IL-25, IL-33, and TSLP, which they suggested were likely to contribute to lung inflammation during different phases of disease progression in COVID-19 [181]. Other targets include type 2 cytokines driven by epithelial alarmin signaling in innate and adaptive immune cells; these could also help treat COVID-19. However, despite their roles in mediating inflammatory responses which contribute to the pathophysiology of severe COVID-19, little work has been conducted to evaluate whether these targets are clinically beneficial in managing severe COVID-19. Therefore, the potential of repurposing existing anti-epithelial alarmin therapies for the management of severe COVID-19 should be investigated.

## 3. Conclusions

In this review, we focused on how epithelial-derived cytokines and chemokines play an important role in perpetuating the hyperinflammatory response and thereby contribute to the pathologies seen in severe forms of COVID-19, such as acute lung injury. This review has built upon the work of Ryu and Shin (2021), whose review emphasized the role of ACE2 and factors that alter its expression within the airway epithelium, and the work of Bridges et al. (2022), whose review considered the responses of the lower airways and gas exchange units to SARS-CoV-2 [182,183]. As discussed, the airway epithelium acts as a sentinel by recognizing markers associated with SARS-CoV-2 infection and subsequently inducing an antiviral response. However, it has been observed that maladaptive epithelial responses can arise in COVID-19, and these can exacerbate the severity of the disease. These responses can influence the behaviour of nearby systems, such as endothelial cells, in an autocrine or paracrine manner. Still, they primarily induce the recruitment and activation of both the innate and adaptive immune systems. The epithelial-derived cytokines and chemokines also have strong potential as biomarkers as they have been observed to be elevated in patients with severe forms of the disease. Some may play a prognostic role in predicting clinical outcomes for patients. Accordingly, epithelial-derived immune mediators also represent targets for therapeutic options, and their study represents an important field of investigation for COVID-19 research.

## Data Availability

Not applicable.
